# Ischemic Stroke in the Course of COVID-19 in a 16-Year-Old Boy

**DOI:** 10.3390/jcm12226963

**Published:** 2023-11-07

**Authors:** Bartłomiej Syzdoł, Anna Maria Rzewuska, Wiktoria Sielwanowska, Monika Żybowska, Natalia Anna Wilczek, Magdalena Maria Woźniak

**Affiliations:** 1 Students’ Scientific Society at the Department of Pediatric Radiology, Medical University of Lublin, 20-059 Lublin, Poland; bartlomiej.syzdol@gmail.com (B.S.); aniarzewuska@wp.pl (A.M.R.); wiktoria.sielwanowska@gmail.com (W.S.); zybowska.m@gmail.com (M.Ż.); 2 Students’ Research Group at the Department of Epidemiology and Clinical Research Methodology, Medical University of Lublin, 20-059 Lublin, Poland; natalia.wilczek09@gmail.com; 3 Department of Pediatric Radiology, Medical University of Lublin, 20-059 Lublin, Poland

**Keywords:** MISC, ischemic stroke, COVID-19 compilations

## Abstract

The SARS-CoV-2 virus that causes COVID-19 disease is still evolving and, despite the end of the acute phase of the pandemic, still poses a risk to public health. One of the very rare complications, occurring in less than 1% of children, is multisystem inflammatory syndrome in children (MISC). Due to the risk of thromboembolic complications as well as cardiac problems, MISC carries a number of life-threatening complications. We report a case of a 16-year-old boy who was hospitalized due to general weakness, fever, conjunctivitis, vomiting and diarrhoea. In view of the mother’s positive result of the SARS-CoV-2 test, the teenager underwent numerous laboratory tests. Taking into account the critical condition of the patient, anticoagulant and antipyretic treatment, steroids and IVIG were added. During hospitalisation, alarming symptoms occurred, including dysarthria, drooping corner of the mouth and muscle weakness on the right side. The magnetic resonance imaging showed changes characteristic of ischemic stroke. Further studies are needed to assess possible thrombotic complications in children after SARS-CoV-2 infection, and specialists should be more vigilant in paediatric patients presenting with such symptoms.

## 1. Introduction

Coronavirus disease 2019 (COVID-19) is caused by severe acute respiratory syndrome coronavirus 2 (SARS-CoV-2). The first cases of this infection were reported in Wuhan in 2019 [1]. Despite reports that the occurrence of COVID-19 in children is less common than in adults, one should remember the dangerous complications of this disease and carefully approach the diagnostic process in case of suspected SARS-CoV-2 infection in children. [2]. COVID-19 in children may manifest itself with a number of symptoms, ranging from asymptomatic course to the development of a severe form of the disease [3]. Dangerous consequences of COVID-19 may include, among others, severe pneumonia, autoinflammatory shock or multisystem inflammatory syndrome associated with COVID-19 (MISC); due to these consequences, they may require hospitalisation and may even need to stay in the Intensive Care Unit (ICU) [4].

MISC is a significant complication of SARS-CoV-2 virus infection and was described by the World Health Organization (WHO) in May 2020 [5]. School-age children are most commonly affected, with the median age of onset occurring at around nine years of age [6]. It is a relatively rare condition that occurs in less than 1% of paediatric patients who have had COVID-19, even when the infection was asymptomatic [6,7]. MISC develops within 3–5 weeks of COVID-19 and is the result of dysregulation of immune system processes [8]. It is characterised by persistent fever and many non-specific symptoms mainly related to the gastrointestinal, respiratory, circulatory and neurological systems. Such symptoms occur in 11–30% of patients [9]. The WHO criteria for the diagnosis of MISC are shown in Table 1.

Symptoms can sometimes be similar to Kawasaki disease, but there are differences in the clinical presentation and the presence of cardiovascular symptoms [11,12]. The disease can carry a number of life-threatening complications, i.e., thromboembolic complications, heart failure and many other less characteristic symptoms; hence, it is an important indication for hospitalisation. Thromboembolism affects an average of 1.4% to 6.5% of paediatric patients who developed MISC, mostly in children aged 12 years and above [13]. Existing reports have shown an increased incidence of ischemic stroke in children in the setting of COVID-19 and MISC. It is estimated that approximately 0.3–0.8% of hospitalized children during SARS-CoV-2 infection may present with an ischemic stroke [14]. Some patients may also develop shock and multiple organ failure [6,7]. Current treatment recommendations include administration of intravenous immunoglobulin (IVIG) and use of glucocorticosteroids (GCS). In severe treatment-resistant cases, biologic drugs are used as well [15]. The in-hospital mortality rate ranges up to about 10% and is slightly higher in children under two years of age [16]. Most paediatric patients recover with resolution of cardiac symptoms and systemic inflammation [15]. We present a case of a 16-year-old boy with several characteristic features of MISC associated with SARS-CoV-2 infection and thromboembolic complications.

## 2. Case Presentation

A 16-year-old boy was admitted to the Paediatric Cardiology Department for suspected MISC. The boy had had fever up to 38.5 °C for 10 days, diarrhoea for 7 days and had been vomiting for 3 days. Two days before hospitalisation, constipation and dysuric symptoms had appeared. The patient’s mother was on home isolation due to a positive test for COVID-19. On admission, the boy was in a critical general condition and there was difficulty in communicating with him. He reported fatigue and was noticeably getting tired when answering questions. Physical examination revealed conjunctivitis, vividly red tongue with white coating, reddened throat, dry mucous membranes, reduced vesicular murmur in the right hemithorax, quiet heart tones, negative meningeal signs, blood pressure 90/45 mmHg and heart rate 80/min. Dysuric symptoms that the boy struggled with two days before admission were related to urinary tract infection (UTI). Table 2 shows laboratory tests performed on admission, and 8, 24, 48 and 72 h after admission.

Cardiac arrhythmias (premature ventricular contraction, type 1 second-degree atrioventricular block, third-degree atrioventricular block) were observed on the electrocardiogram. An echocardiographic study was performed, where slightly reduced left and right ventricular ejection fraction was found. The examination revealed no heart defects.

A central venous catheter was inserted into the right common jugular vein. Crystalloids, calcium, antithrombin III, anticoagulants (acetylsalicylic acid—ASA), antipyretics and antibiotic therapy (ceftriaxone, and gentamicin) were administered. After initiating an infusion of catecholamines (dobutamine, and dopamine), systemic steroid therapy was administered as well as IVIG due to persistent febrile conditions. In the following days, the current treatment was maintained, and the boy’s condition improved. 

**Figure 1:**
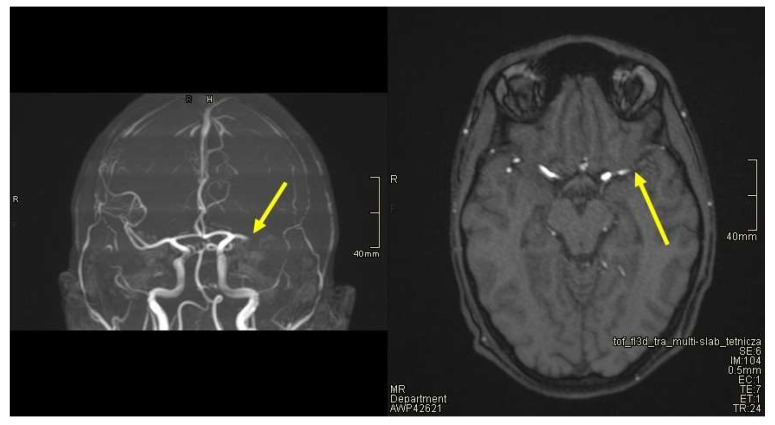
A thrombus was visualized in the lumen of the distal part of the M1 segment of the left middle cerebral artery. Perfusion deficits were identified in the M2 segment and distal branches of the left middle cerebral artery. Yellow arrows represent described changes.

**Figure 2:**
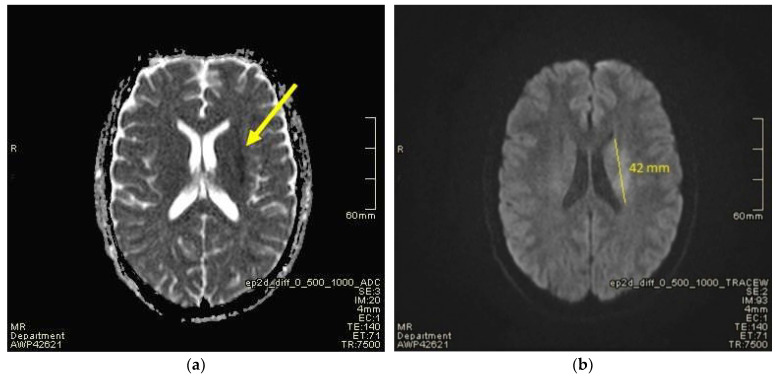
Ischemic strokes involving the corona radiata and lenticular nuclei on the left side. Measurement of AP (anteroposterior) dimension was about 42 mm—changes visible only in the ADC (**a**) and DWI (**b**) sequence. Yellow arrow (**a**) and measurement line (**b**) represent described changes.

Suddenly, during the administration of IVIG in the evening on the third day of hospitalisation, the boy developed dysarthria and drooping of the corner of the mouth on the right side. Dysarthria is defined as a speech disorder resulting from injury of the motor component of the motor-speech system and is characterized by poor articulation or pronunciation of phonemes and words. The patient involuntarily urinated, and he was sleepy and confused. On examination, there was muscle weakness of the right upper and lower limb, absence of tendon reflexes and central paresis of the VII nerve on the right side. Meningeal signs were negative. Cultivation tests revealed no bacteria in blood culture. Due to the suspicion of ischaemic stroke, magnetic resonance imaging (MRI) of the head was performed together with MRI angiography (Figure 1 and Figure 2).

A thrombectomy was performed. During the following days of hospitalisation, coagulation parameters normalised, and echocardiographic examination showed improvement of the left and right ventricular ejection fraction. A complete neurological examination revealed slight paresis of the VII nerve on the right side. The patient was discharged from the department after 19 days of hospitalisation. A complete neurological examination 6 months after the stroke revealed no abnormal findings.

## 3. Discussion

The patient was admitted with general weakness, fever, conjunctivitis, vomiting and diarrhoea. The symptoms presented by the patient, the laboratory findings (including significantly elevated inflammatory parameters), and the direct contact with his mother suffering from COVID-19 facilitated the diagnosis of multisystem inflammatory syndrome in children (MISC). The patient met the criteria for diagnosis of the disease published by the WHO, which were listed in the introduction of this article. As mentioned before, MISC takes the form of multiple organ failure, which is also presented in this case report. 

Thromboembolic complications such as ischemic stroke are not common complications of COVID-19 infection and are particularly rare in children [14,17]. In the current case, ischemic stroke involved corona radiata and lenticular nuclei on the left side. The thrombus was visualized in the lumen of the distal part of the M1 segment of the left MCA. In the case report of Carney et al. about a 3-year-old boy meeting MISC criteria, treated IVIG, ASA and methylprednisolone on the second day of hospitalisation, the patient experienced sudden weakness in the right arm and leg, aphasia and overall neurologic deficit. MRI showed evidence of restricted diffusion consistent with ischemia of the territory of the middle division of the left MCA [18]. In the present case cranial nerve VII dysfunction was observed. In another case of a 10-year-old girl in the neurological examinations, ipsilateral cranial nerve VII dysfunction was observed. MRI showed an acute infarction in the right putamen, globus pallidus, and the posterior part of the insula. A small single focal dilatation within the M1 segment of the MCA was also observed [19]. In the present case, report inflammatory state parameters were highly elevated. In another study, a 12-year-old boy with dysarthria, aphasia and right hemiparesis MRI of the brain showed a small acute border-zone infarction in the left MCA territory. A rapid COVID-19 test was positive. However, inflammatory state parameters were within the norm and D-Dimer was not obtained [20]. The correlation between the occurrence of ischemic stroke in the course of MISC is not fully understood and further diagnosis of this correlation is needed. There are reports of prothrombotic properties of SARS-CoV-2 virus. Activation of the coagulation system and damage to vascular endothelial cells may contribute to thrombus formation and the development of ischaemic stroke [21]. Testing for SARS-CoV-2 should be considered for all children with ischaemic stroke. There are publications that show an increase in ischaemic stroke cases since the COVID-19 pandemic began [22]. As thromboembolic complications are among the most serious, there have been recent studies that report positive effects of low-dose ASA (3–5 mg/kg) in MISC. ASA has anti-inflammatory and antiplatelet effects and may therefore reduce the incidence of thromboembolic complications [23]. The cardiovascular system is one of the most common systems that is affected in the course of MISC. The pathomechanism is not well understood and it is suspected that a cytokine storm is mainly responsible [24]. Nevertheless, there is a significant likelihood that the activation of inflammatory processes and alterations in endothelial cells plays a pivotal role in the development of MISC. COVID-19 illness infection triggers the activation of T lymphocytes, subsequently activating immune pathways involving macrophages, monocytes, plasma cells, and B cells. These mechanisms collectively contribute to a “cytokine storm” and the onset of MISC. Furthermore, reduced numbers of NK cells have been observed in MISC cases, which may also correlate with the development of this condition [25]. Replication of SARS-CoV-2 leads to an increase in the level of pro-inflammatory substances such as interferon-γ and interleukins IL-1β and IL-6. As a result, endothelial cells sustain damage, leading to inflammation and the activation of platelets [26]. Activated vascular endothelial cells secrete adhesion molecules such as VCAM-1, ICAM-1, and E-selectin, which in turn trigger the activation of immune cells. In this process, activated leukocytes interact with blood platelets, leading to the formation of microthrombi and subsequent secretion of pro-inflammatory molecules [27]. Another theory involves the expression of angiotensin converting enzyme 2 (ACE2). These receptors are found, among others, in the lungs, intestines, muscles, liver, pancreas, kidneys, in addition to arterial and vascular endothelium [28]. The conjunction of the virus particle to the ACE2 receptor causes a decrease in the activity of the receptor, which in turn leads to an increase in the concentration of angiotensin II. Angiotensin II activates intracellular pathways that lead to blood vessel constriction, cell damage, inflammatory processes and increased oxidative stress [29]. Myocardial cells also express the ACE2 receptor, to which SARS-CoV-2 has increased affinity. Their involvement and destruction occur in approximately 20% of all patients infected with SARS-CoV-2 [30,31]. Cardiovascular involvement was also present in our patient—echocardiography showed a reduction in left and right ventricular ejection fraction.

Treatment of MISC syndrome is mainly based on intravenous immunoglobulin (1–2 g/kg) and low to medium doses of glucocorticosteroids. High doses of glucocorticosteroids in the form of pulses of methylprednisolone are only used in patients with life-threatening conditions. Some patients, if fever persists, require a second dose of immunoglobulin infusion [32]. If treatment with GCS and IVIG is not effective, biologic drugs such as tocilizumab, infliximab or anakinra can be used. Another therapeutic option is the use of plasma from recovered patients. Due to the documented thromboembolic risk, low doses of ASA (3–5 mg/kg) are used [33]. It is presumed that vaccination can prevent the occurrence of MISC. A 2022 cohort study in Denmark showed that among 52 patients aged 0–17 years, as many as 51 were unvaccinated against COVID-19. The same study also estimated the efficacy of two doses of the Pfizer-BioNTech vaccine, which was the first vaccine approved for use in children. Its efficacy against MISC in the aforementioned study was 96% [34]. Similar vaccine efficacy data was published by US researchers in 2022, who estimated it at 91% [35]. However, there are assumptions that the incidence of MISC in the paediatric patient population may change due to exposure to SARS-CoV-2 to subsequent variants of the virus, especially Omicron, and at the same time due to the increasing vaccination of children. There are reports suggesting that the Omicron variant is less frequently associated with the occurrence of MISC, compared to other variants of the SARS-CoV-2 virus [36,37,38,39]. However, with the emergence of new variants of SARS-CoV-2, further research is needed on the incidence of MISC after different variants of SARS-CoV-2 to clarify whether MISC is indeed a vaccine-preventable disease.

Due to the range of different COVID-19 complications that develop in the course of MISC, a full panel of tests, including blood morphology, inflammatory parameters, D-dimer, electrolytes as well as assessment of renal and myocardial function should be performed in any paediatric patient suspected of developing a multisystem inflammatory syndrome. Among the imaging tests, cardiac echocardiography is extremely important to detect pathology within the myocardium or coronary vessels. A chest X-ray and abdominal ultrasound, if the clinical picture requires it, are also extremely important examinations [40].

## 4. Conclusions

MISC is undoubtedly one of the most dangerous complications following COVID-19. Increased vigilance by specialists is recommended for any paediatric patient with fever and deteriorating general condition that may indicate the development of MISC. It is essential in such cases to take a thorough look into patient’s history during which special attention should be paid to the history of SARS-CoV-2 infection. Specialists should have increased vigilance and awareness of its consequences such as thromboembolic complications resulting in stroke.

## Figures and Tables

**Table 1:** World Health Organization (WHO) criteria for MISC [10].

WHO Criteria for Multisystem Inflammatory Syndrome in Children
Children and adolescents 0–19 years of age with fever ≥ 3 days
AND two of the following:
Rash or bilateral non-purulent conjunctivitis or muco-cutaneous inflammation signs (oral, hands or feet). Hypotension or shock. Features of myocardial dysfunction, pericarditis, valvulitis, or coronary abnormalities (including ECHO findings or elevated Troponin/NT-proBNP). Evidence of coagulopathy (by PT, PTT, elevated d-Dimers). Acute gastrointestinal problems (diarrhoea, vomiting, or abdominal pain).
AND
elevated markers of inflammation such as Erythrocyte Sedimentation Rate (ESR), C-reactive protein, or procalcitonin.
AND
no other obvious microbial cause of inflammation, including bacterial sepsis, staphylococcal or streptococcal shock syndromes.
AND
evidence of COVID-19 (RT-PCR, antigen test or serology positive), or likely contact with patients with COVID-19.

**Table 2:** Patient’s laboratory results.

	On Admission	After 8 h	After 24 h	After 48 h	After 72 h	Reference Range	Unit
RBC	4.18	N/A	4.9	N/A	5.11	4.32–5.75	mln/µL
Hb	12.7	N/A	14.6	N/A	15.5	13.5–17.5	g/dL
MCV	85.9	N/A	84.9	N/A	86.5	81.2–95.1	fl
Platelets	134	N/A	203	N/A	286	140–420	K/µL
CRP	15	17.85	11.05	8.88	5.26	0–0.5	mg/dL
Procalcitonin	3.590	5.560	1.920	1.080	0.692	<0.5	mg/mL
LDH	318	N/A	N/A	N/A	N/A	0–266	U/L
D-dimer	6046	9157	4191	N/A	N/A	<500	ng/mL
CK	490	624	N/A	N/A	N/A	0–270	U/L
CK-MB	41.5	45.2	N/A	N/A	N/A	0–25	U/L
Creatine	1.16	1.67	0.72	N/A	0.73	0.7–1.2	mg/dL
Urea	81.9	92.4	42.8	N/A	36.7	18–45	mg/dL
NT-proBNP	14823	29415	N/A	N/A	4647	0–125	pg/mL
cTnI	14.65	6.16	1.83	1.32	0.62	<0.04	ng/mL
APTT	40.6	45.7	N/A	N/A	N/A	25.4–36.9	s
Fibrinogen	5.94	6.75	N/A	N/A	N/A	1.9–4.0	g/L
Antithrombin III	46	89	91	N/A	N/A	83–128	%
Calcium	1.96	2.00	2.04	2.02	1.95	2.1–2.55	mmoL/L
SARS-CoV-2, IgM	4.01	N/A	N/A	N/A	N/A	<1.1	BAU/mL
SARS-CoV-2, IgG	529	N/A	N/A	N/A	N/A	<33.8	BAU/mL

RBC—red blood cells; Hb—haemoglobin; MCV—mean corpuscular volume; CRP—C-reactive protein; LDH—lactate dehydrogenase; CK—creatine-kinase; CK-MB—creatine kinase muscle and brain isoenzyme; NT-proBNP—N-terminal pro-B-type natriuretic peptide; cTnI—cardiac troponin I; APTT—activated partial thromboplastin time; N/A—not applicable.

## Data Availability

Not applicable.
